# Comparative analysis of restraint stress-induced depressive-like phenotypes in C57BL/6N mice derived from three different sources

**DOI:** 10.1186/s42826-020-00062-0

**Published:** 2020-08-26

**Authors:** Dong-Joo Hwang, Ki-Chun Kwon, Dae-Youn Hwang, Min-Soo Seo, Kil-Soo Kim, Young-Suk Jung, Joon-Yong Cho

**Affiliations:** 1 Exercise Biochemistry Laboratory, Korea National Sport University, Seoul, Republic of Korea; 2 Department of Biomaterials Science, College of Natural Resources and Life Science/Life and Industry Convergence Research Institute, Pusan National University, Busan, Republic of Korea; 3 Laboratory Animal Center, Daegu-Gyeongbuk Medical Innovation Foundation, Daegu, Korea; 4 College of Veterinary Medicine, Kyungpook National University, Daegu, Korea; 5 College of Pharmacy, Pusan National University, Busan, Korea

**Keywords:** C57BL/6NKorl mice, Depressive disorder, Restraint stress

## Abstract

C57BL/6NKorl mice are a novel mouse stock recently developed by the National Institute of Food and Drug Safety Evaluation in Korea. Extensive research into the nature of C57BL/6NKorl mice is being conducted. However, there is no scientific evidence for the phenotypic response to restraint stress (RST), a stress paradigm for modeling depressive disorders, in rodents. In this study, we investigated the repeated RST-induced depressive-like phenotypes in C57BL/6 N mouse substrains (viz., C57BL/6NKorl mice from Korea, C57BL/6NA mice from the United States, and C57BL/6NB mice from Japan) obtained from different sources. The results showed that C57BL/6 N mice derived from various sources exposed to repeated RST resulted in depressive-like phenotypes reflected by a similar degree of behavioral modification and susceptibility to oxidative stress in a duration-dependent manner, except for the distinctive features (increased body weight (BW) and tolerance to the suppression of BW gain by exposure to repeated RST) in C57BL/6NKorl mice. Taken together, the duration-dependent alteration in depressive-like phenotypes by repeated exposure to RST observed in this study may provide valuable insights into the nature of C57BL/6NKorl mice as an alternative animal resource for better understanding of the etiology of depressive disorders and the mechanisms of antidepressant actions.

## Introduction

Depression is a mental disorder characterized by psychological symptoms such as continuous low mood or sadness, diminished interest or pleasure in activities, anxiety, and social life disability. The prevalence of depression and depressive-like symptoms has been increasing in recent decades [1, 2]. Various causative risk factors have been reported to increase the risk of depressive disorder, but its pathophysiology is not fully understood. Recently, to better understand the etiology of depressive disorder and the mechanisms of antidepressant action, different paradigms have been evaluated in relation to the validity, replicability, and molecular insight related to depressive disorder [1, 3, 4].

Meanwhile, it has been reported that chronic stress can precipitate a psychiatric disorder with increased susceptibility to developing anxiety- and depressive-like phenotypes [3]. Exposure to chronic stress is associated with an imbalance in neurotransmitters and negative feedback on the activation of the hypothalamic-pituitary-adrenal axis, which in turn induces adrenal secretion of glucocorticoids, specifically corticosterone (CORT) in rodents, and cortisol in humans [5, 6]. The neuroendocrine system responds to stress that underlies depressive disorders. In this respect, there is an increasing interest in using a restraint stress (RST) paradigm to recapitulate the features of depressive disorders in rodents [7, 8]. Immobilization induced by restraint constituting physical interference has been shown to promote extensive epigenetic modification of neural networks in the brain, thereby leading to depressive-like behaviors, as determined by the state of behavioral despair and anhedonia [9–11].

In rodents, the RST-induced depression model involving movement restriction for at least 2 h a day for several days has produced significant progress toward understanding the mechanisms underlying depressive-like behaviors that closely resemble those of patients with depression. However, it has been suggested that exposure to chronic stress has a different impact on behavior and neurochemical responses depending on the biological characteristics such as strain and the gender of rodents [11, 12]. These findings provide a rational basis for research on such animals based on a detailed understanding of genetic backgrounds and phenotypic properties.

C57BL/6NKorl is a novel mouse stock recently developed from the C57BL/6 strain background, the most prevalent strain of inbred mice, by the Korean National Institute of Food and Drug Safety Evaluation (NIFDS) [13, 14]. Growing evidence indicates that, with some distinctive features primarily related to body weight (BW), the responses of C57BL/6NKorl mice to various chemicals and physiological stimuli (e.g., MPTP administration, methionine and choline-deficient diet feeding, and fertilization) are similar to other mice of different origins (the United States and Japan) supplied by commercial vendors [14–16]. However, there is no evidence base for repeated RST-induced depressive-like phenotypes, especially in C57BL/6NKorl mice.

Therefore, this study aimed to investigate the repeated RST-induced depressive-like phenotypes in C57BL/6 N mouse origins (viz., C57BL/6NKorl mice from Korea, C57BL/6NA mice from the United States, and C57BL/6NB mice from Japan) derived from different sources. A better understanding of the responses to repeated RST in mice will provide scientific evidence to ensure the proper application of these C57BL/6 N mice as a useful laboratory animal resource.

## Materials and methods

### Animals

All experimental procedures were approved by the Institutional Animal Care and Use Committee of the Korean National Sport University (Approval Number: KNSU-IACUC-2019-04). Male C57BL/6 N mice (7-weeks old, 21–24 g) were obtained from three different sources (C57BL/6NKorl mice were provided by the Department of Laboratory Animal Resources at NIFDS (Cheongju, Korea), C57BL/6NA and C57BL/6NB mice were purchased from Orient Bio Inc. (Gyeonggi-do, Korea), and Japan SLC Inc. (Shizuoka, Japan), respectively. Upon arrival, the three types of mice (C57BL/6NKorl, C57BL/6NA, and C57BL/6NB) were randomly divided into four groups [Control (*n* = 10), restraint stress-treated group for 7 days (RST 7d, *n* = 10), restraint stress-treated group for 14 days (RST 12d, *n* = 10), and restraint stress-treated group for 21 days (RST 21d, *n* = 10), and then housed in controlled pathogen-free laboratory conditions (12:12 h dark-light cycle, 20 ± 1 °C, and 50% relative humidity) with ad libitum access to standard chow diet and water at the laboratory animal facility of the Korean National Sport University.

### Experimental design

To induce the animal model of depressive disorder, RST was selected as a stress paradigm considering previous studies and results drawn from PubMed keyword (depressive disorder, mouse strain, among others) searching, experimental procedures, and behavioral analogs for depression symptoms reflected by paradigm [17]. The RST exposure for the experimental animals was performed by individually placing each animal in a 50 ml polyethylene tube modified with reference to the previous studies to provide ventilation so that immobilization can be maintained. RST of 2 h per day was performed for 7, 14, or 21 days, starting at 10 a.m. Experimental animals exposed on the same day were transferred to a dedicated cage to receive a standard chow diet and water.

### Biological features

The BW changes of experimental animals induced by exposure to repeated RST was measured periodically at the scheduled times, and a body composition analysis was precisely measured using dual-energy X-ray absorptiometry before euthanasia of the experimental animals. The measurement parameters consisted of body mass (g), fat mass (g), lean mass (g), and fat ratio (% Fat).

### Behavioral assessments

In the forced swim test (FST), mice were individually placed into a cylinder of tap water (23–25 °C) for 5 min of habituation and then returned to their home cage after drying. After 24 h, the same procedures were conducted as a test trial for 6 min. The test session was recorded, and the time spent was measured by dividing the animal’s behavior into three types: immobility, swimming, and climbing. Immobility time was used as the main parameter for representative depressive-like behavior patterns. To exclude the ceiling effect due to excessive struggling and rather than floating in the early stages of FST, the first minute was excluded from the results [18].

The tail suspension test (TST) was performed as previously described. Briefly, mice were individually suspended by adhesive tape attached to a 1 cm section of the tail tip in a position 50 cm above the ground. The 6 min test session was recorded, and the time spent immobile, indicative of depressive-like behavior, was measured. As with the FST, to exclude the ceiling effect due to excessive struggling in the early stages of TST, the first minute was excluded from the results [19].

For the open field test (OFT), the levels of activity and anxiety of experimental animals exposed to brightly lit open areas, as indications of anxiety- and depressive-like behaviors, were measured using zone analysis. The open field apparatus (40 × 40 × 30 cm) was fabricated from white acrylic with a floor divided into 16 squares, with the center four squares set as the center zone and the remaining 12 squares as the peripheral zone. The measurement of the degree of exploration activity of the experimental animals was conducted for 5 min, and the time spent in the central and peripheral regions, the number of entries from the peripheral zone to the central zone, and line crossing were measured using zone analysis [20].

### Enzyme-linked immunosorbent assays

Serum corticosterone (CORT) levels were analyzed quantitatively using a corticosterone ELISA assay kit (Enzo Life Sciences, ADI-900-097) according to the manufacturer’s instructions. Briefly, to prepare a standard curve, the standard diluent was dispensed into appropriate wells. Afterward, 100 μL of the samples diluted with steroid-displacement reagent solution were dispensed into new wells and incubated with antibody reagent on a plate shaker for 2 h, followed by serial standard protocols. The optical density was read at 405 nm on a microplate reader (HIDEX, Turku, Finland). The CORT concentration was calculated from a standard curve fit.

Lipid peroxidation was determined by the reaction of MDA with thiobarbituric acid (TBA) to form a colorimetric product. MDA levels were analyzed using a commercial assay kit (Sigma Aldrich, MAK085) according to the manufacturer’s instructions. First, a standard curve fit was prepared using MDA standard solutions of different concentrations, and the samples containing butylhydroxytoluene were incubated at 95 °C for 60 min to form an MDA-TBA adduct, and then cooled to room temperature in an ice bath for 10 min. The reaction absorbance at 532 nm was measured using a microplate reader (HIDEX, Turku, Finland). The MDA concentration for samples was calculated from a standard curve fit, and then expressed as nanomoles per milliliter.

### Immunoblotting

Extracted brain tissue was homogenized with RIPA lysis buffer (EBA-1149, ELPISBIOTECH, Korea) using a portable homogenizer, and quantified using the BCA protein assay. The sample was electrophoresed on a 12% SDS-polyacrylamide gel with 30 μg of total protein, transferred to a nitrocellulose membrane, blocked with 10% bovine serum albumin, and reacted for 24 h at 4 °C with primary antibody: 4-hydroxynonenal (4-HNE, Abcam, diluted 1:1000). The following day, the membrane was incubated with horseradish peroxidase (HRP)-conjugated secondary antibody for 4 h at room temperature. Then each membrane was reacted with western blotting luminol reagent solution (SC-2048, Santa Cruz Biotechnology, USA) for 2 min. The immunoblots and densitometric analyses were performed using an image analysis system (Molecular Imager ChemiDoc XRS System, Bio-Rad, USA) and quantitated by Quantity One 1-D Analysis Software (Bio-Rad, USA). The quantification of multiple bands detected by Anti-4-HNE antibody (predicted molecular weight, 20–80 kDa) was carried out by normalizing the immunoreactive band densities to Ponseau S reactive bands.

### Immunohistochemistry

After cardio perfusion with 4% PFA, fixed brain tissue was serially sectioned at a thickness of 35 μm by cryosection method using a freezing microtome and stained using the free-floating method. To retrieve the antigen masked by formalin-fixation, the brain tissue sections were immersed in a culture dish containing 0.01 M sodium citrate for 40 min in a water bath at 80 °C. After that, the samples were washed three times with PBS for 5 min then blocked with 1% normal serum at room temperature for 60 min. The tissue samples were incubated with primary anti-tryptophan hydroxylase (TPH) antibody (EDM Millipore, AB1541, diluted 1:200) at 4 °C overnight. The following day, the sections were incubated with biotinylated goat anti-rabbit secondary antibody for 4 h, followed by color development with diaminobenzidine (DAB, Vector, USA). Images of stained brain tissue were obtained using an optical microscope (Leica, Nussloch, Germany).

### Statistics

All data were analyzed using GraphPad PRISM 8 software (GraphPad Software. Inc., CA, USA). The statistical significance of differences in measured variables among groups was analyzed using a one-way analysis of variance (ANOVA) followed by the Bonferroni post hoc test for multiple comparisons. Data were expressed as the mean ± standard error (SEM), and statistical significance was tested at *p* < 0.05.

## Results

### Effect of repeated RST on body weight gain and body composition

**Fig. 1:**
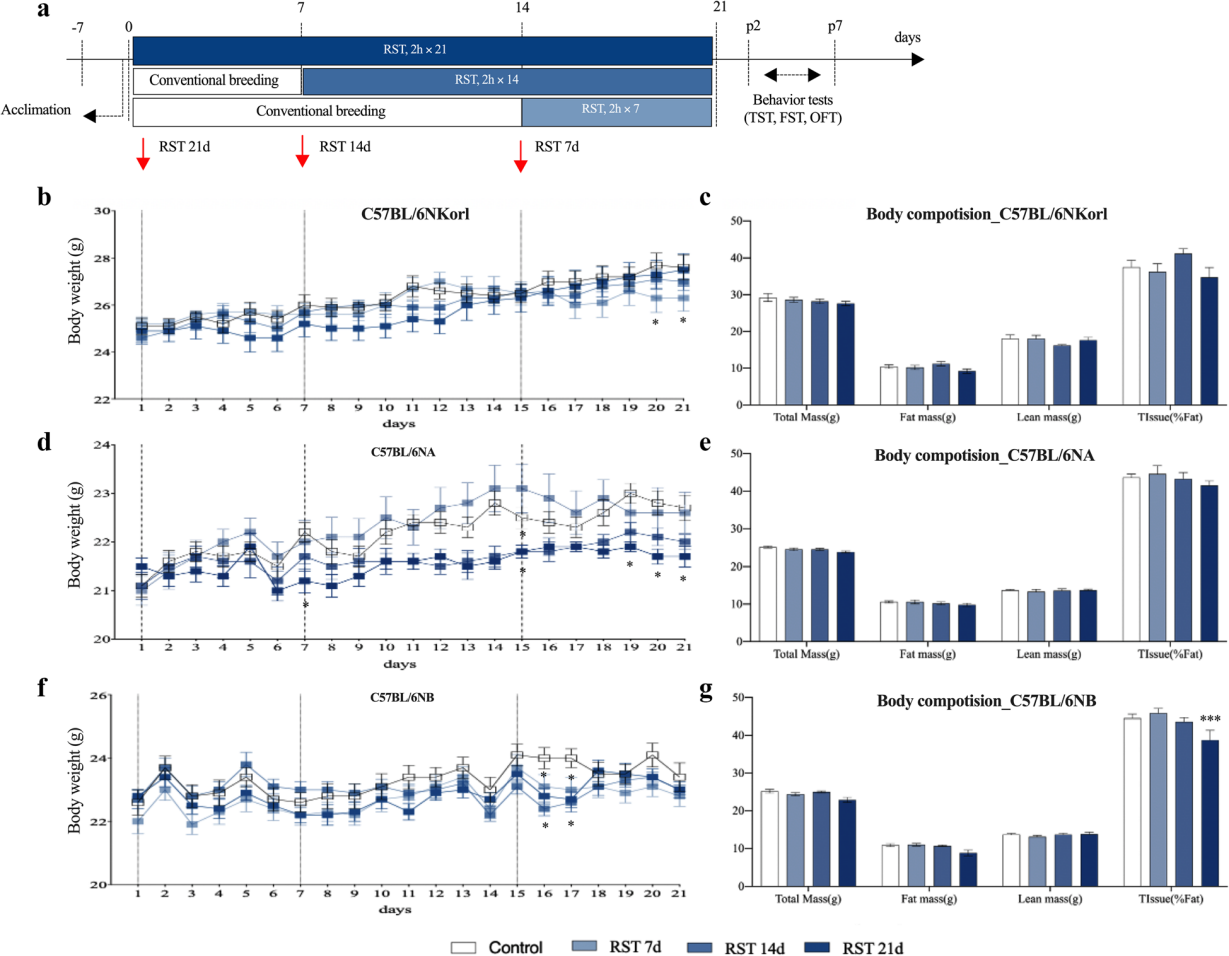
Effect of repeated RST treatment on C57BL/6 N mice body weight changes. **(a)** Schematic of the experimental design. (**b**, **d**, and **f**) The suppression of body weight gain and (**c**, **e**, and **g**) body composition (total mass, fat mass, lean mass, and fat ratio) in C57BL/6NKorl, C57BL/6NA, and C57BL/6NB mice exposed to repeated RST. Data are presented as the mean ± SEM; *n* = 10 mice per group. *Denotes statistical difference from the control mice (*p < 0.05; ***p < 0.001). RST, restraint stress; SEM, mean ± standard error

Extensive research has suggested the progressive suppression of BW gain induced by exposure to repeated RST as one of the important indicators for the diagnosis of depressive disorder. We first monitored the changes in BW of mice exposed to repeated RST for 7, 14, and 21 consecutive days (Fig. 1). At the starting time point (8-weeks old), C57BL/6NKorl mice had the highest initial BW compared to C57BL/6NA and C57BL/6NB mice (Fig. 1). Notably, C57BL/6NA and C57BL/6NB mice showed a duration-dependent suppression in BW gain following exposure to repeated RST, whereas C57BL/6NKorl mice were found to have the lowest BW in mice exposed to RST for 7 days, indicating a tolerance (from the time point of 14 days or later) to stress-induced suppression of BW gain after 14 days of RST treatment (Fig. 1). These results suggest that C57BL/6NKorl mice possess a distinctive feature regarding BW changes with relatively higher BW and adaptive response to exposure to repeated RST.

We further examined the changes in body composition of mice 9 days after the last RST exposure. Following RST exposure, C57BL/6NKorl, C57BL/6NA, and C57BL/6NB mice showed a trend towards a reduction in BW, with loss of fat mass implying that reduction of fat mass in body composition is a major factor contributing to the loss of BW caused by repeated RST (Fig. 1).

### Repeated RST-induced anxiety- and depressive-like behaviors

**Fig. 2:**
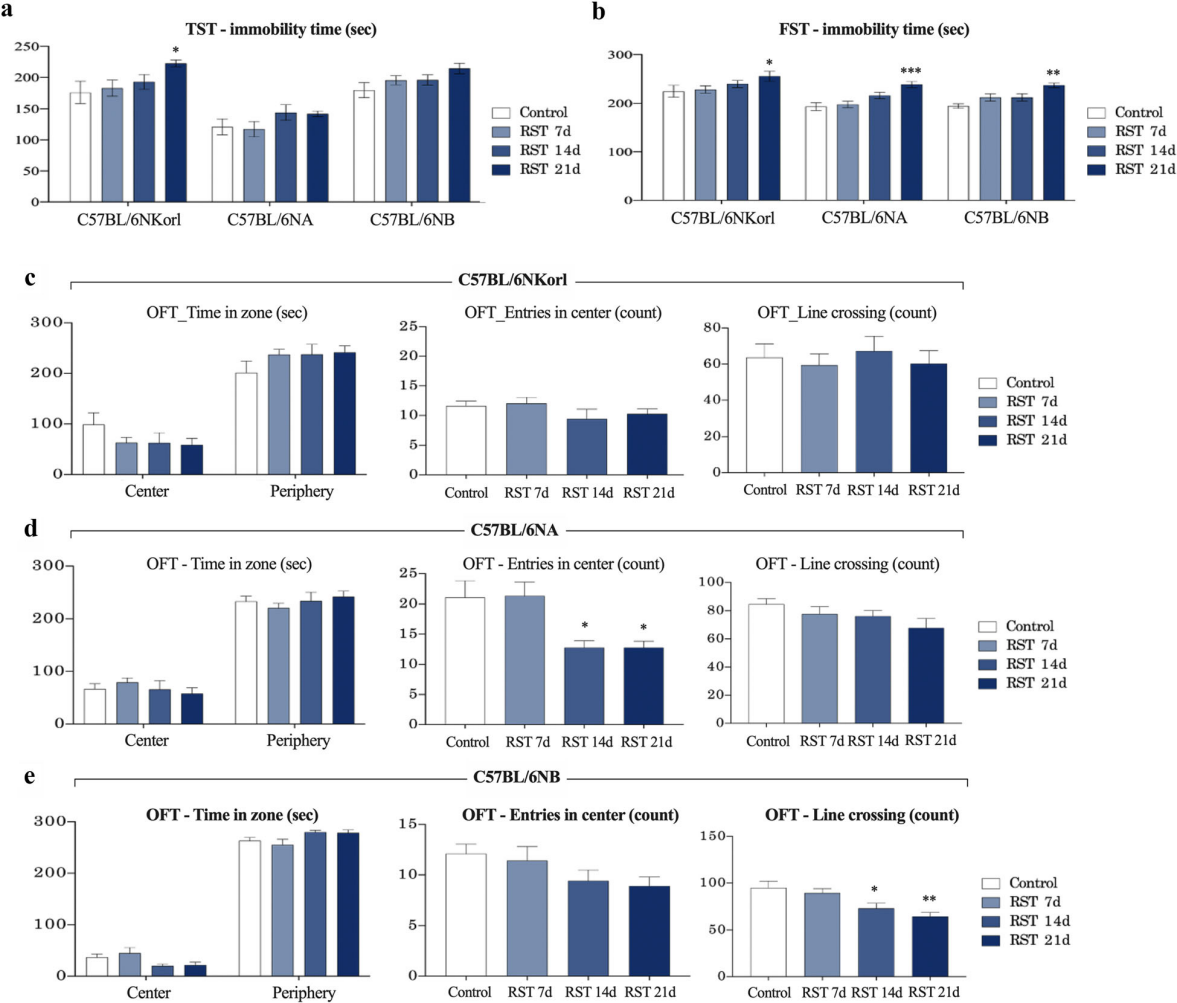
Repeated RST-induced anxiety- and depressive-like behaviors in mice derived from three different sources. (**a**, **b**) Immobility time in FST and TST, and **c**, **d**, and **e** The time spent in the center and periphery zones, entries in the center, and the number of line crossings in the zone analysis of OFT following exposure to repeated restraint stress. Data are presented as the mean ± SEM; *n* = 10 mice per group. *Denotes statistical difference from the control mice (*p < 0.05; **p < 0.01; ***p < 0.001). RST, restraint stress, FST, forced swim test; TST, tail suspension test; OFT, open field test; SEM, mean ± standard error

As an indicator for determining the depressive disorder shown in mice exposed to RST, we utilized multiple behavioral tests to assess anxiety- and depressive-like behaviors (Fig. 2). As expected, in the tail suspension test (TST) and the forced swim test (FST), C57BL/6NKorl, C57BL/6NA, and C57BL/6NB mice showed a tendency for linearly decreased times spent immobile, depending on the duration of RST exposure. Much more immobility time was observed in mice exposed to RST for 21 days compared with control mice (Fig. 2). Similarly, in the zone analysis of the open field test (OFT), C57BL/6NKorl, C57BL/6NA, and C57BL/6NB mice revealed a decrease in the magnitude of activity and anxiety-like behaviors depending on the duration of RST treatment, as shown in the trend towards decreased time spent in the center zone, entries to the center, the number of line crossings, and increased times spent in the periphery zone. However, most of these behavioral changes did not reach statistical significance (Fig. 2). These results imply that C57BL/6 N mice derived from different sources exhibit overall similar depressive-like behaviors following RST exposure in a duration-dependent manner.

### CORT and lipid peroxidation responses to repeated RST

**Fig. 3:**
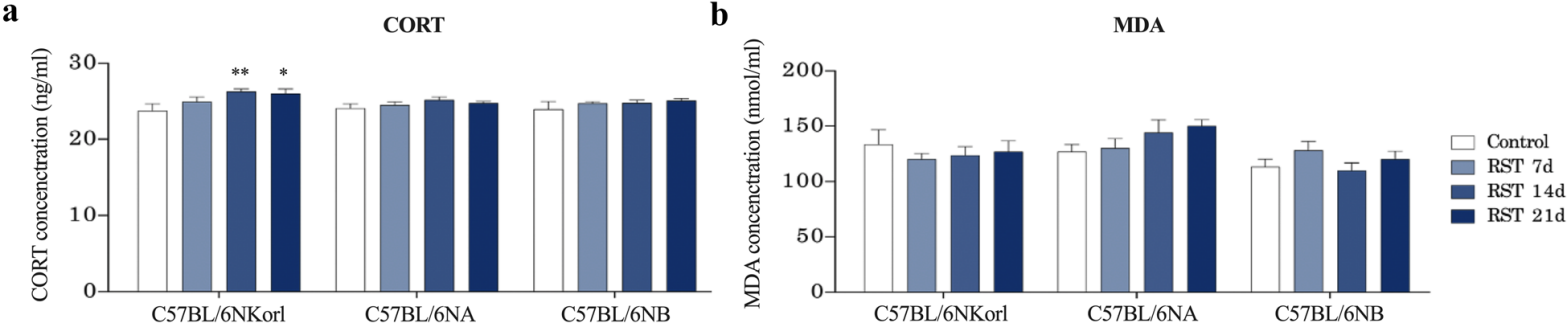
Effect of repeated RST treatment on CORT and MDA levels in mice from different sources. (**a**) Changes in CORT and (**b**) MDA levels in mice exposed to repeated RST. Data are presented as the mean ± SEM; *n* = 6 mice per group. *Denotes statistical difference from the control mice (*p < 0.05; **p < 0.01). RST, restraint stress; CORT, corticosterone; SEM, mean ± standard error

We examined the RST-induced stress response in the blood by measuring CORT and lipid peroxidation (MDA) levels (Fig. 3). C57BL/6NKorl mice showed significantly increased CORT levels following exposure to repeated RST for more than 14 days. Conversely, C57BL/6NA and C57BL/6NB mice were not significantly different, implying that C57BL/6NKorl mice are more sensitive to stress compared with the other mouse stocks (Fig. 3). Conversely, no significant difference was observed in MDA levels, with a few variations.

### Repeated RST-induced oxidative stress

**Fig. 4:**
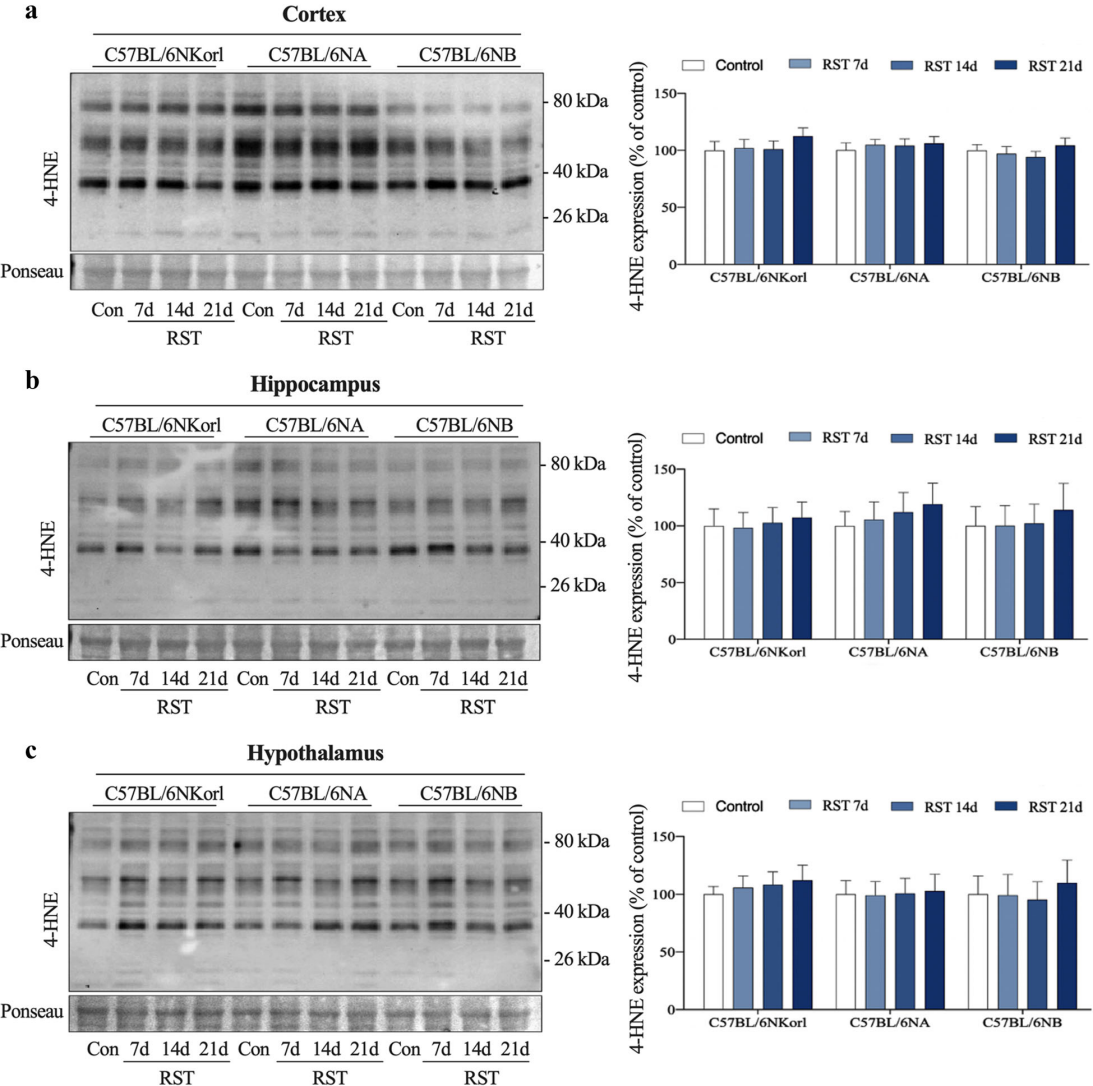
The effect of repeated RST treatment on 4-HNE expression in different brain regions. Representative immunoblots and densitometric analysis of 4-HNE expression relative to the control group in (**a**) cortical, (**b**) hippocampal, and (**c**) hypothalamic homogenates. Ponceau S was used as a loading control. Data are presented as the mean ± SEM; *n* = 4 mice per group. RST, restraint stress; 4-HNE, 4-hydroxynonenal; SEM, mean ± standard error

We examined whether restraint stress-induced depressive-like phenotypes are accompanied by cellular oxidative stress responses by measuring the expression levels of 4-Hydroxynoneal (4-HNE) in different brain regions (hippocampus, cortex, and hypothalamus) of mice exposed to RST (Fig. 4). C57BL/6NKorl, C57BL/6NA, and C57BL/6NB mice showed a trend towards an increase in 4-HNE expression in three different brain regions, although it did not reach statistical significance. These results suggest that repeated RST exposure is implicated in neurological oxidative damage in the brain nervous system.

### The expression pattern of TPH in mice exposed to repeated RST

**Fig. 5:**
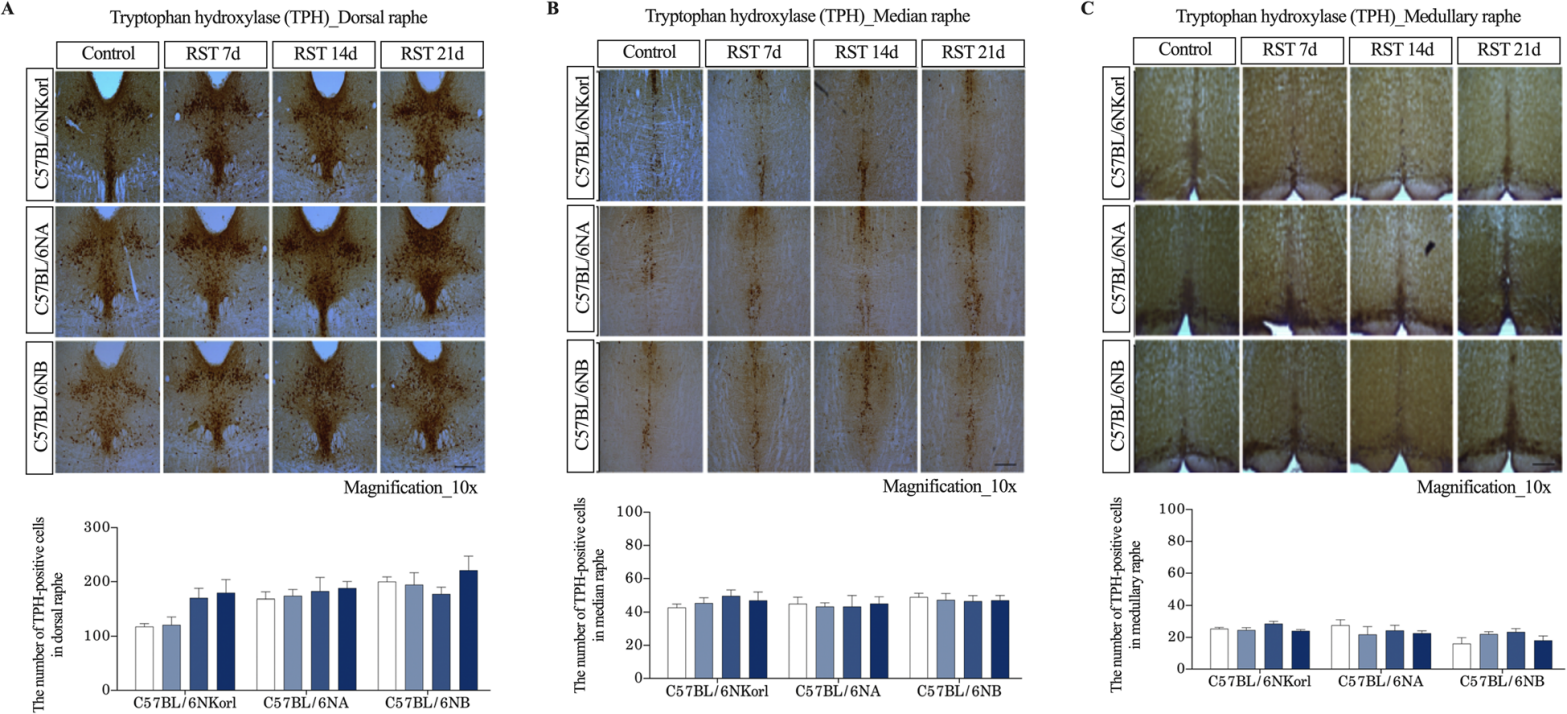
Effect of repeated RST treatment on histological expression pattern of TPH-positive neurons in raphe nuclei. Representative histological images and quantification of TPH-positive cells in (**a**) the dorsal raphe nucleus (DRN) and (**b**) the median raphe nucleus (MRN), and (**c**) the medullary raphe nucleus (10x magnification). TPH-positive cells were identified as brown granular dots. Results are expressed as TPH^+^ cell number per section. Scale bar = 250 μm. Data are presented as the mean ± SEM; *n* = 4 mice per group. RST, restraint stress; TPH, tryptophan hydroxylase; SEM, mean ± standard error

Finally, we quantitatively evaluated the expression of TPH, which is involved in the biosynthesis of serotonin (5-Hydroxytryptamine, 5-HT) in brain regions (raphe nuclei) of mice exposed to repeated RST by performing immunohistochemical staining (Fig. 5). Quantification of TPH-positive cells showed an increasing trend in the dorsal raphé nucleus (DRN, 4.5–4.7 mm posterior from bregma) of C57BL/6NKorl, C57BL/6NA, and C57BL/6NB mice, depending on the RST exposure duration. However, the difference was not statistically significant (Fig. 5). However, no specific changes were observed in the median (MRN, 4.5–4.7 mm posterior from bregma) and medullary regions (rMRN, 5.5–6.2 mm posterior from bregma) of the raphé nucleus. These results suggest that C57BL/6NKorl, C57BL/6NA, and C57BL/6NB mice have a similar neurobiological response to repeated RST exposure in the DNR region.

## Discussion

In our study, we established an animal model of a depressive disorder by periodic exposure to repeated RST for different durations (7, 14, or 21 consecutive days) to compare the different effects in C57BL/6 N mice derived from various sources. This stress paradigm mimics phenotypes resembling those of patients with depressive disorder.

In our results, we found that repeated RST suppressed BW gain in all mice depending on the duration of RST exposure, except for the distinctive feature of C57BL/6NKorl mice showing tolerance (from the time point of 14 days or later) to stress-induced suppression of BW gain with much higher BW. Based on previous studies that observed BW changes after repeated RST treatment over 14 days in rodents, these are very unusual results, suggesting that the Korl-specific genetic background may also contribute to phenotypic differences, especially for body weight gain [11, 13, 21]. The mechanism of BW changes by repeated RST remains to be elucidated. In this study, we confirmed that a reduction in fat mass in body composition is a major factor contributing to the loss of BW through body composition analysis 7 days after the last exposure to repeated RST. It was previously reported that a reduction in body fat could be attributed to the secretion of glucocorticoids (GC), which stimulates brown adipose tissue (BAT), which is involved in thermoregulation [21, 22]. Thus, it was deduced that the stress-related BAT-derived thermogenic effect could have contributed to the distinctive patterns of BW-related features seen in C57BL/6NKorl mice.

Upon exposure to repeated RST, all mice exhibited the expected depressive-like behaviors with at least 14 days of exposure to RST, as reflected by prolonged immobility, decreased locomotion, and thigmotactic responses (the time spent in the periphery) in the TST, FST, and OFT, all of which are widely used for assessing anxiety- and depressive-like behaviors in research using rodents [18–20]. These results are consistent with previous studies [17, 23, 24] which demonstrated adaptive behavioral changes, suggesting that C57BL/6 N mice derived from different sources develop a similar degree of depressive-like behaviors upon exposure to repeated RST.

Changes in CORT levels are likely to be predictive of susceptibility to stress stimuli [5]. C57BL/6NA and C57BL/6NB mice showed no change (a tendency toward subtle increase), while C57BL/6NKorl mice showed significantly increased CORT levels when exposed to RST for more than 14 days. Based on a previous study reporting that elevated CORT levels returned to steady-state levels around 7 days after cessation of RST [5, 9]. Although we did not measure an acute adaptive CORT response, our results, which were measured 7 days after the cessation of RST indicate that basal CORT levels of C57BL/6NKorl mice were elevated by exposure to chronic stress, implying that C57BL/6NKorl mice have a higher susceptibility to exposure to chronic stress compared with other mice. Furthermore, we found that, as reflected by a pattern of relatively increased 4-HNE expression, a reactive lipid peroxidation end product, repeated RST induced an enhanced accumulation of reactive oxygen species (ROS) in brain sub-regions (the hippocampus, cortex, and hypothalamus) of mice exposed to RST for 21 days. However, levels did not reach statistical significance. These results indicate that stress may cause extensive oxidative damage by a redox imbalance of the brain’s nervous system [25, 26], suggesting that mice derived from different sources have similar susceptibility to oxidative stress upon exposure to repeated RST.

Tryptophan hydroxylase (TPH) is a rate-limiting enzyme responsible for the biosynthesis of 5-HT, which is a predisposing factor for depression [27]. There are several conflicting studies on the expression pattern of TPH following exposure to stress. Still, it has been reported that the levels of 5-HT and TPH in the raphe nuclei of the brain stem are tightly linked to the development of a depressive disorder or depressive-like behaviors [28–30]. In the present study, although not statistically significant, all mice exposed to repeated RST showed a trend towards increased TPH expression selectively in the dorsal raphe nucleus, the largest serotonergic nucleus, depending on the duration of RST exposure. Increased TPH expression was not observed in the median raphe or medullary raphe nuclei. This finding is in agreement with previous studies using RST treatment as a stressor to induce depressive disorder, which reported the elevation of TPH expression [29, 30]. Confirmatory evidence is lacking, but our findings suggest that repeated RST may selectively activate serotonergic neurons with a compensatory mechanism regulated explicitly in the dorsal raphe for deficient serotonin.

The limitation of this study was that it did not provide a specific basis for the distinctive features (the much higher BW and the tolerance to the suppression of BW gain by exposure to repeated RST) of BW in C57BL/6NKorl mice, and the results that did not reach statistical significance were interpreted based on this tendency. Therefore, further studies should be performed to provide a scientific basis for the characterization of C57BL/6NKorl mice with an extensive understanding of the stress responses of mice derived from different sources.

## Conclusion

In this study, we found that exposure to repeated RST in C57BL/6 N mice derived from three different sources (viz., C57BL/6NKorl mice from Korea, C57BL/6NA mice from the United States, and C57BL/ 6NB mouse from Japan) resulted in depressive-like phenotypes reflected by a similar degree of behavioral modification and susceptibility to oxidative stress, along with different patterns in the suppression of BW gain in C57BL/6NKorl mice. Taken together, the duration-dependent alteration in depressive-like phenotypes by exposure to repeated RST in this study may provide valuable insight into the nature of C57BL/6NKorl mice as an alternative animal resource for better understanding the etiology of depressive disorder and mechanisms of antidepressant action.

## Data Availability

The authors confirm that the data supporting the findings of this study are available within the article.

## Abbreviations

4-HNE: 4-hydroxynonenal
BW: Body weight
CORT: Corticosterone
FST: Forced swim test
NIFDS: Korean National Institute of Food and Drug Safety Evaluation
RST: Restraint stress
OFT: Open field test
TST: Tail suspension test
5-HT: Serotonin (5-Hydroxytryptamine)
TBA: Thiobarbituric acid
